# 7-(4-Chloro­benzyl­idene)-3-[(4-chloro­phen­oxy)meth­yl]-6-(4-nitro­thio­phen-2-yl)-7*H*-1,2,4-triazolo[3,4-*b*][1,3,4]thia­diazine

**DOI:** 10.1107/S1600536811015637

**Published:** 2011-04-29

**Authors:** Hoong-Kun Fun, Safra Izuani Jama Asik, Ibrahim Abdul Razak, Nithinchandra, Balakrishna Kalluraya

**Affiliations:** a X-ray Crystallography Unit, School of Physics, Universiti Sains Malaysia, 11800 USM, Penang, Malaysia; b Department of Studies in Chemistry, Mangalore University, Mangalagangotri, Mangalore 574 199, India

## Abstract

In the title compound, C_22_H_13_Cl_2_N_5_O_3_S_2_, the thia­diazine ring adopts a half-chair conformation. The benzene rings of the chloro­phen­oxy and chloro­benzyl groups and the thio­phene ring form dihedral angles of 35.6 (1), 80.7 (1) and 14.2 (1)°, respectively, with the triazole ring. In the crystal, mol­ecules are connected into sheets parallel to (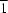
11) by inter­molecular C—H⋯N and C—H⋯Cl hydrogen bonds. In addition, π–π stacking inter­actions are observed between thio­phene and triazole rings, and between inversion-related triazole rings [centroid–centroid distances = 3.5975 (11) and 3.4324 (11) Å].

## Related literature

For general background to and applications of 1,2,4-triazole derivatives, see: Shujuan *et al.* (2004 ▶); Clemons *et al.* (2004 ▶); Johnston (2002 ▶); Wei *et al.* (2007 ▶). For ring conformations and ring puckering analysis, see: Cremer & Pople (1975 ▶). For bond-length data, see: Allen *et al.* (1987 ▶); Jin *et al.* (2004 ▶). For related structures, see: Goh *et al.* (2010**a* ▶,*b* ▶,*c* ▶,d* ▶).
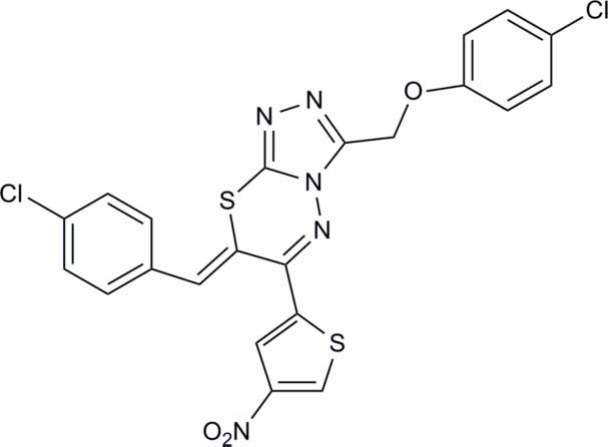


## Experimental

### 

#### Crystal data

C_22_H_13_Cl_2_N_5_O_3_S_2_
*M* *_r_* = 530.39
Triclinic, 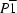

*a* = 8.5021 (2) Å
*b* = 10.0379 (2) Å
*c* = 14.3623 (3) Å
α = 94.434 (1)°
β = 97.981 (1)°
γ = 109.242 (1)°
*V* = 1136.07 (4) Å^3^
*Z* = 2
Mo *K*α radiation
μ = 0.51 mm^−1^
*T* = 296 K
0.39 × 0.32 × 0.11 mm

#### Data collection

Bruker SMART APEXII CCD area-detector diffractometer
Absorption correction: multi-scan (*SADABS*; Bruker, 2009 ▶) *T*_min_ = 0.827, *T*_max_ = 0.946
19590 measured reflections
6548 independent reflections
5142 reflections with *I* > 2σ(*I*)
*R*_int_ = 0.031

#### Refinement

*R*[*F* ^2^ > 2σ(*F* ^2^)] = 0.044
*wR*(*F* ^2^) = 0.133
*S* = 1.04
6548 reflections
307 parameters
H-atom parameters constrained
Δρ_max_ = 0.59 e Å^−3^
Δρ_min_ = −0.46 e Å^−3^

### 

Data collection: *APEX2* (Bruker, 2009 ▶); cell refinement: *SAINT* (Bruker, 2009 ▶); data reduction: *SAINT*; program(s) used to solve structure: *SHELXTL* (Sheldrick, 2008 ▶); program(s) used to refine structure: *SHELXTL*; molecular graphics: *SHELXTL*; software used to prepare material for publication: *SHELXTL* and *PLATON* (Spek, 2009 ▶).

## Supplementary Material

Crystal structure: contains datablocks global, I. DOI: 10.1107/S1600536811015637/ci5185sup1.cif

Structure factors: contains datablocks I. DOI: 10.1107/S1600536811015637/ci5185Isup2.hkl

Additional supplementary materials:  crystallographic information; 3D view; checkCIF report

## Figures and Tables

**Table 1:** Hydrogen-bond geometry (Å, °)

*D*—H⋯*A*	*D*—H	H⋯*A*	*D*⋯*A*	*D*—H⋯*A*
C15—H15*A*⋯N2^i^	0.93	2.60	3.495 (3)	162
C21—H21*A*⋯Cl1^ii^	0.93	2.81	3.691 (2)	159

Symmetry codes: (i) 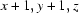; (ii) 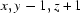.

**Fig. 1.**
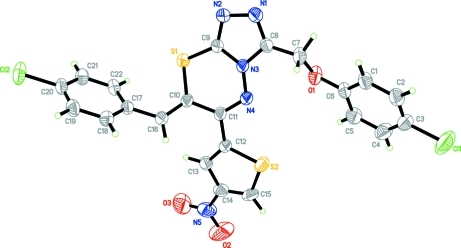
The molecular structure of the title compound, showing 50% probability displacement ellipsoids and the atom-numbering scheme.

**Fig. 2.**
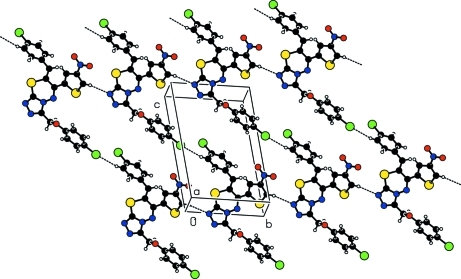
The crystal packing of the title compound, viewed along the a axis. Hydrogen bonds are shown as dashed lines.

## supplementary crystallographic information

### Comment

The 1,2,4-triazole nucleus has been incorporated into a wide variety of therapeutically interesting compounds. Several compounds containing 1,2,4-triazole rings are well known as drugs. For example, fluconazole is used as an antimicrobial drug (Shujuan *et al.*, 2004), while vorozole, letrozole and anastrozole are non-steroidal drugs used for the treatment of cancer (Clemons *et al.*, 2004) and loreclezole is used as an anticonvulsant (Johnston *et al.*, 2002). Similarly substituted derivatives of triazole possess comprehensive bioactivities such as antimicrobial, anti-inflammatory, analgesic, antihypertensive, anticonvulsant and antiviral activities (Wei *et al.*, 2007). In continuation of our search on the synthesis of biologically active compounds, we synthesized triazolothiadiazine from triazole.

In the title compound, the 1,2,4-triazole (C8/N1–N3/C9) and thiophene (C12–C15/S2) rings are essentially planar, with maximum deviations of 0.006 (2) and 0.003 (2) Å for atom C9 and C14, respectively. The 1,3,4-thiadiazine (C9–C11/N3/N4/S1) ring is slightly distorted and may be regarded as having a half-chair conformation with puckering parameters, Q = 0.474 (2) Å, θ = 113.9 (2)°, φ = 149.1 (2)° (Cremer & Pople, 1975).

The two benzene rings (C1–C6 and C17–C22) and the thiophene (C12–C15/S2) ring form dihedral angles of 35.6 (1), 80.7 (1) and 14.2 (1)°, respectively, with the 1,2,4-triazole ring (C8/N1–N3/C9). The geometric parameters are consistent with those observed in closely related structures (Goh *et al.*, 2010**a*,*b*,*c*,d*). The bond lengths show normal values (Allen *et al.* 1987).

In the crystal packing (Fig. 2), the molecules are connected by intermolecular C15—H15A···N2 and C21—H21A···Cl1 interactions that link the molecules into two–dimensional arrays parallel to the (1 1 1). In addition, the molecular packing is also stabilized by π–π stacking interactions between thiophene (C12–C15/S2; centroid *Cg*1) and 1,2,4-triazole (C8/N1–N3/C9; centroid *Cg*2) rings, with a *Cg*1···*Cg*2^#^ separation of 3.5975 (11) Å (symmetry code #: 1-x, 1-y, -z), and that between 1,2,4-triazole (C8/N1–N3/C9) rings at (x, y, z) and (-x, 1-y, -z), with their centroids separated by 3.4324 (11) Å.

### Experimental

To a soultion of 4-Amino-5-[(*p*-chlorophenoxy)methyl]-4*H*-1,2,4-triazole-3-thiol (0.01 mol) and 2-bromo-3-(*p*-chlorophenyl)-1-(5-nitrothiophen-2-yl) prop-2-en-1-one (0.01 mol) in ethanol, a catalytic amount of anhydrous sodium acetate was added. The solution was refluxed on a water bath for 9 h. The solid product that separated out was filtered and dried. It was then recrystallized from ethanol. Single crystals suitable for X-ray analysis were obtained from a 1:2 mixture of DMF and ethanol by slow evaporation.

### Refinement

H atoms were placed in calculated positions with C–H = 0.93–0.97 Å. The *U*_iso_ value of H atoms were constrained to be 1.2*U*_eq_ of the carrier atom.

### Crystal data

**Table:**

C_22_H_13_Cl_2_N_5_O_3_S_2_	*Z* = 2
*M**_r_* = 530.39	*F*(000) = 540
Triclinic, *P*1	*D*_x_ = 1.551 Mg m^−^^3^
Hall symbol: -P 1	Mo *K*α radiation, λ = 0.71073 Å
*a* = 8.5021 (2) Å	Cell parameters from 5767 reflections
*b* = 10.0379 (2) Å	θ = 2.7–32.0°
*c* = 14.3623 (3) Å	µ = 0.51 mm^−^^1^
α = 94.434 (1)°	*T* = 296 K
β = 97.981 (1)°	Plate, yellow
γ = 109.242 (1)°	0.39 × 0.32 × 0.11 mm
*V* = 1136.07 (4) Å^3^	

### Data collection

**Table:**

Bruker SMART APEXII CCD area-detector diffractometer	6548 independent reflections
Radiation source: fine-focus sealed tube	5142 reflections with *I* > 2σ(*I*)
graphite	*R*_int_ = 0.031
φ and ω scans	θ_max_ = 30.0°, θ_min_ = 2.4°
Absorption correction: multi-scan (*SADABS*; Bruker, 2009)	*h* = −11→11
*T*_min_ = 0.827, *T*_max_ = 0.946	*k* = −14→14
19590 measured reflections	*l* = −20→20

### Refinement

**Table:**

Refinement on *F*^2^	Primary atom site location: structure-invariant direct methods
Least-squares matrix: full	Secondary atom site location: difference Fourier map
*R*[*F*^2^ > 2σ(*F*^2^)] = 0.044	Hydrogen site location: inferred from neighbouring sites
*wR*(*F*^2^) = 0.133	H-atom parameters constrained
*S* = 1.04	*w* = 1/[σ^2^(*F*_o_^2^) + (0.0647*P*)^2^ + 0.3979*P*] where *P* = (*F*_o_^2^ + 2*F*_c_^2^)/3
6548 reflections	(Δ/σ)_max_ = 0.001
307 parameters	Δρ_max_ = 0.59 e Å^−^^3^
0 restraints	Δρ_min_ = −0.46 e Å^−^^3^

### Special details

**Table:**

Geometry. All esds (except the esd in the dihedral angle between two l.s. planes) are estimated using the full covariance matrix. The cell esds are taken into account individually in the estimation of esds in distances, angles and torsion angles; correlations between esds in cell parameters are only used when they are defined by crystal symmetry. An approximate (isotropic) treatment of cell esds is used for estimating esds involving l.s. planes.
Refinement. Refinement of F^2^ against ALL reflections. The weighted R-factor wR and goodness of fit S are based on F^2^, conventional R-factors R are based on F, with F set to zero for negative F^2^. The threshold expression of F^2^ > 2sigma(F^2^) is used only for calculating R-factors(gt) etc. and is not relevant to the choice of reflections for refinement. R-factors based on F^2^ are statistically about twice as large as those based on F, and R- factors based on ALL data will be even larger.

### Fractional atomic coordinates and isotropic or equivalent isotropic displacement parameters (Å^2^)

**Table:**

	*x*	*y*	*z*	*U*_iso_*/*U*_eq_	
S1	0.31310 (6)	0.39243 (5)	0.13806 (3)	0.03764 (12)	
S2	0.65367 (7)	0.90408 (5)	0.02701 (4)	0.04462 (14)	
Cl1	0.32206 (12)	1.05741 (9)	−0.46034 (7)	0.0906 (3)	
Cl2	0.17004 (8)	0.36669 (8)	0.58962 (4)	0.06035 (17)	
N1	0.0052 (2)	0.34827 (18)	−0.09684 (11)	0.0418 (4)	
N2	0.0543 (2)	0.29741 (17)	−0.01333 (11)	0.0391 (3)	
N3	0.23458 (18)	0.51207 (16)	−0.01635 (10)	0.0316 (3)	
N4	0.37735 (19)	0.63329 (16)	0.00403 (10)	0.0349 (3)	
N5	1.0471 (2)	0.9832 (2)	0.22741 (17)	0.0602 (5)	
O1	0.25195 (18)	0.62527 (17)	−0.20276 (10)	0.0451 (3)	
O2	1.1570 (3)	1.0856 (3)	0.2079 (2)	0.0994 (8)	
O3	1.0661 (3)	0.9215 (3)	0.29489 (15)	0.0825 (6)	
C1	0.1175 (3)	0.7481 (2)	−0.31056 (13)	0.0416 (4)	
H1A	0.0097	0.6926	−0.3027	0.050*	
C2	0.1376 (3)	0.8504 (2)	−0.37127 (14)	0.0455 (5)	
H2A	0.0436	0.8644	−0.4045	0.055*	
C3	0.2982 (3)	0.9316 (2)	−0.38211 (16)	0.0486 (5)	
C4	0.4404 (3)	0.9153 (3)	−0.33232 (18)	0.0540 (5)	
H4A	0.5479	0.9717	−0.3399	0.065*	
C5	0.4199 (3)	0.8139 (2)	−0.27120 (15)	0.0464 (5)	
H5A	0.5145	0.8028	−0.2364	0.056*	
C6	0.2593 (2)	0.7284 (2)	−0.26134 (12)	0.0367 (4)	
C7	0.0975 (3)	0.5693 (2)	−0.16998 (14)	0.0433 (4)	
H7A	0.0089	0.5158	−0.2227	0.052*	
H7B	0.0664	0.6464	−0.1426	0.052*	
C8	0.1140 (2)	0.4746 (2)	−0.09737 (12)	0.0355 (4)	
C9	0.1912 (2)	0.39706 (18)	0.03185 (12)	0.0321 (3)	
C10	0.4010 (2)	0.57962 (18)	0.16945 (12)	0.0314 (3)	
C11	0.4557 (2)	0.66196 (18)	0.09118 (12)	0.0317 (3)	
C12	0.6138 (2)	0.78458 (19)	0.10904 (12)	0.0336 (3)	
C13	0.7497 (2)	0.8140 (2)	0.17988 (13)	0.0379 (4)	
H13A	0.7535	0.7623	0.2307	0.045*	
C14	0.8840 (2)	0.9339 (2)	0.16526 (15)	0.0416 (4)	
C15	0.8518 (3)	0.9942 (2)	0.08699 (16)	0.0456 (5)	
H15A	0.9278	1.0739	0.0687	0.055*	
C16	0.4132 (2)	0.64263 (19)	0.25710 (12)	0.0351 (4)	
H16A	0.4627	0.7413	0.2673	0.042*	
C17	0.3573 (2)	0.57331 (19)	0.33897 (12)	0.0336 (3)	
C18	0.2834 (3)	0.6385 (2)	0.40089 (13)	0.0405 (4)	
H18A	0.2732	0.7258	0.3906	0.049*	
C19	0.2251 (3)	0.5746 (2)	0.47743 (13)	0.0435 (4)	
H19A	0.1746	0.6180	0.5179	0.052*	
C20	0.2426 (2)	0.4462 (2)	0.49313 (12)	0.0392 (4)	
C21	0.3189 (3)	0.3808 (2)	0.43452 (13)	0.0411 (4)	
H21A	0.3314	0.2947	0.4463	0.049*	
C22	0.3765 (2)	0.4453 (2)	0.35809 (12)	0.0377 (4)	
H22A	0.4290	0.4021	0.3188	0.045*	

### Atomic displacement parameters (Å^2^)

**Table:**

	*U*^11^	*U*^22^	*U*^33^	*U*^12^	*U*^13^	*U*^23^
S1	0.0452 (3)	0.0336 (2)	0.0311 (2)	0.01036 (19)	0.00304 (18)	0.00748 (16)
S2	0.0439 (3)	0.0399 (3)	0.0487 (3)	0.0095 (2)	0.0095 (2)	0.0183 (2)
Cl1	0.1026 (6)	0.0671 (4)	0.1053 (6)	0.0239 (4)	0.0179 (5)	0.0532 (4)
Cl2	0.0592 (4)	0.0895 (4)	0.0412 (3)	0.0276 (3)	0.0205 (2)	0.0311 (3)
N1	0.0374 (9)	0.0467 (9)	0.0348 (8)	0.0072 (7)	0.0040 (6)	0.0042 (7)
N2	0.0395 (9)	0.0373 (8)	0.0343 (8)	0.0054 (7)	0.0059 (6)	0.0041 (6)
N3	0.0280 (7)	0.0361 (7)	0.0276 (6)	0.0057 (6)	0.0063 (5)	0.0072 (5)
N4	0.0309 (7)	0.0381 (8)	0.0310 (7)	0.0043 (6)	0.0068 (6)	0.0083 (6)
N5	0.0348 (10)	0.0588 (12)	0.0699 (14)	0.0003 (9)	0.0002 (9)	−0.0045 (10)
O1	0.0376 (7)	0.0657 (9)	0.0394 (7)	0.0221 (7)	0.0121 (6)	0.0219 (6)
O2	0.0397 (10)	0.0808 (15)	0.144 (2)	−0.0193 (10)	−0.0013 (12)	0.0271 (14)
O3	0.0505 (11)	0.1096 (17)	0.0642 (12)	0.0057 (11)	−0.0134 (9)	0.0150 (11)
C1	0.0357 (10)	0.0531 (11)	0.0356 (9)	0.0142 (9)	0.0050 (7)	0.0100 (8)
C2	0.0512 (12)	0.0492 (11)	0.0379 (10)	0.0227 (10)	0.0005 (8)	0.0052 (8)
C3	0.0614 (14)	0.0369 (10)	0.0472 (11)	0.0146 (9)	0.0109 (10)	0.0109 (8)
C4	0.0462 (12)	0.0482 (12)	0.0640 (14)	0.0089 (10)	0.0117 (10)	0.0162 (10)
C5	0.0370 (10)	0.0531 (12)	0.0483 (11)	0.0151 (9)	0.0045 (8)	0.0099 (9)
C6	0.0361 (9)	0.0473 (10)	0.0274 (8)	0.0153 (8)	0.0051 (7)	0.0060 (7)
C7	0.0338 (10)	0.0613 (12)	0.0345 (9)	0.0139 (9)	0.0063 (7)	0.0156 (8)
C8	0.0308 (9)	0.0439 (10)	0.0287 (8)	0.0090 (7)	0.0048 (6)	0.0046 (7)
C9	0.0328 (8)	0.0338 (8)	0.0285 (7)	0.0086 (7)	0.0076 (6)	0.0050 (6)
C10	0.0283 (8)	0.0348 (8)	0.0291 (8)	0.0071 (7)	0.0062 (6)	0.0073 (6)
C11	0.0302 (8)	0.0349 (8)	0.0295 (8)	0.0083 (7)	0.0086 (6)	0.0075 (6)
C12	0.0316 (9)	0.0332 (8)	0.0328 (8)	0.0049 (7)	0.0087 (7)	0.0074 (6)
C13	0.0331 (9)	0.0427 (10)	0.0329 (8)	0.0063 (8)	0.0058 (7)	0.0053 (7)
C14	0.0309 (9)	0.0404 (10)	0.0457 (10)	0.0031 (8)	0.0069 (8)	0.0002 (8)
C15	0.0408 (11)	0.0304 (9)	0.0591 (12)	0.0004 (8)	0.0172 (9)	0.0059 (8)
C16	0.0346 (9)	0.0352 (9)	0.0315 (8)	0.0056 (7)	0.0082 (7)	0.0046 (7)
C17	0.0302 (8)	0.0395 (9)	0.0274 (8)	0.0073 (7)	0.0052 (6)	0.0037 (6)
C18	0.0448 (11)	0.0387 (9)	0.0372 (9)	0.0115 (8)	0.0118 (8)	0.0053 (7)
C19	0.0466 (11)	0.0532 (12)	0.0327 (9)	0.0178 (9)	0.0138 (8)	0.0031 (8)
C20	0.0331 (9)	0.0540 (11)	0.0263 (8)	0.0086 (8)	0.0047 (7)	0.0089 (7)
C21	0.0434 (11)	0.0495 (11)	0.0341 (9)	0.0201 (9)	0.0051 (8)	0.0118 (8)
C22	0.0389 (10)	0.0496 (10)	0.0278 (8)	0.0192 (8)	0.0060 (7)	0.0055 (7)

### Geometric parameters (Å, °)

**Table:**

S1—C9	1.7342 (18)	C5—C6	1.386 (3)
S1—C10	1.7727 (18)	C5—H5A	0.93
S2—C15	1.694 (2)	C7—C8	1.486 (3)
S2—C12	1.7330 (17)	C7—H7A	0.97
Cl1—C3	1.736 (2)	C7—H7B	0.97
Cl2—C20	1.7386 (18)	C10—C16	1.339 (2)
N1—C8	1.302 (2)	C10—C11	1.483 (2)
N1—N2	1.403 (2)	C11—C12	1.467 (2)
N2—C9	1.303 (2)	C12—C13	1.364 (3)
N3—C9	1.364 (2)	C13—C14	1.413 (3)
N3—C8	1.376 (2)	C13—H13A	0.93
N3—N4	1.383 (2)	C14—C15	1.354 (3)
N4—C11	1.296 (2)	C15—H15A	0.93
N5—O3	1.210 (3)	C16—C17	1.468 (2)
N5—O2	1.221 (3)	C16—H16A	0.93
N5—C14	1.449 (3)	C17—C22	1.391 (3)
O1—C6	1.375 (2)	C17—C18	1.397 (3)
O1—C7	1.410 (2)	C18—C19	1.384 (3)
C1—C2	1.382 (3)	C18—H18A	0.93
C1—C6	1.391 (3)	C19—C20	1.377 (3)
C1—H1A	0.93	C19—H19A	0.93
C2—C3	1.378 (3)	C20—C21	1.381 (3)
C2—H2A	0.93	C21—C22	1.383 (3)
C3—C4	1.381 (3)	C21—H21A	0.93
C4—C5	1.378 (3)	C22—H22A	0.93
C4—H4A	0.93		
			
C9—S1—C10	95.62 (8)	N3—C9—S1	121.40 (13)
C15—S2—C12	92.18 (10)	C16—C10—C11	122.26 (16)
C8—N1—N2	107.87 (15)	C16—C10—S1	122.39 (13)
C9—N2—N1	106.29 (15)	C11—C10—S1	115.32 (12)
C9—N3—C8	104.63 (14)	N4—C11—C12	114.57 (15)
C9—N3—N4	128.75 (14)	N4—C11—C10	125.55 (16)
C8—N3—N4	126.23 (14)	C12—C11—C10	119.85 (15)
C11—N4—N3	115.80 (14)	C13—C12—C11	128.47 (16)
O3—N5—O2	124.2 (2)	C13—C12—S2	111.57 (14)
O3—N5—C14	118.5 (2)	C11—C12—S2	119.62 (13)
O2—N5—C14	117.2 (2)	C12—C13—C14	110.48 (17)
C6—O1—C7	115.44 (15)	C12—C13—H13A	124.8
C2—C1—C6	119.61 (19)	C14—C13—H13A	124.8
C2—C1—H1A	120.2	C15—C14—C13	115.29 (18)
C6—C1—H1A	120.2	C15—C14—N5	122.83 (19)
C3—C2—C1	119.4 (2)	C13—C14—N5	121.8 (2)
C3—C2—H2A	120.3	C14—C15—S2	110.47 (15)
C1—C2—H2A	120.3	C14—C15—H15A	124.8
C2—C3—C4	121.7 (2)	S2—C15—H15A	124.8
C2—C3—Cl1	119.05 (18)	C10—C16—C17	127.24 (17)
C4—C3—Cl1	119.29 (18)	C10—C16—H16A	116.4
C5—C4—C3	118.8 (2)	C17—C16—H16A	116.4
C5—C4—H4A	120.6	C22—C17—C18	118.34 (16)
C3—C4—H4A	120.6	C22—C17—C16	122.56 (16)
C4—C5—C6	120.4 (2)	C18—C17—C16	119.10 (17)
C4—C5—H5A	119.8	C19—C18—C17	120.80 (18)
C6—C5—H5A	119.8	C19—C18—H18A	119.6
O1—C6—C5	116.08 (17)	C17—C18—H18A	119.6
O1—C6—C1	123.84 (17)	C20—C19—C18	119.36 (18)
C5—C6—C1	120.07 (18)	C20—C19—H19A	120.3
O1—C7—C8	109.98 (16)	C18—C19—H19A	120.3
O1—C7—H7A	109.7	C19—C20—C21	121.20 (17)
C8—C7—H7A	109.7	C19—C20—Cl2	119.24 (15)
O1—C7—H7B	109.7	C21—C20—Cl2	119.55 (16)
C8—C7—H7B	109.7	C20—C21—C22	119.05 (18)
H7A—C7—H7B	108.2	C20—C21—H21A	120.5
N1—C8—N3	109.93 (16)	C22—C21—H21A	120.5
N1—C8—C7	124.21 (17)	C21—C22—C17	121.21 (17)
N3—C8—C7	125.52 (17)	C21—C22—H22A	119.4
N2—C9—N3	111.26 (15)	C17—C22—H22A	119.4
N2—C9—S1	127.32 (14)		
			
C8—N1—N2—C9	−0.1 (2)	C16—C10—C11—N4	−140.1 (2)
C9—N3—N4—C11	−21.5 (3)	S1—C10—C11—N4	38.2 (2)
C8—N3—N4—C11	166.81 (17)	C16—C10—C11—C12	42.0 (3)
C6—C1—C2—C3	0.0 (3)	S1—C10—C11—C12	−139.59 (14)
C1—C2—C3—C4	−1.3 (3)	N4—C11—C12—C13	−156.04 (19)
C1—C2—C3—Cl1	178.71 (16)	C10—C11—C12—C13	22.0 (3)
C2—C3—C4—C5	0.8 (4)	N4—C11—C12—S2	16.7 (2)
Cl1—C3—C4—C5	−179.26 (18)	C10—C11—C12—S2	−165.26 (13)
C3—C4—C5—C6	1.1 (4)	C15—S2—C12—C13	−0.15 (16)
C7—O1—C6—C5	161.28 (18)	C15—S2—C12—C11	−174.03 (15)
C7—O1—C6—C1	−19.9 (3)	C11—C12—C13—C14	173.10 (18)
C4—C5—C6—O1	176.5 (2)	S2—C12—C13—C14	−0.1 (2)
C4—C5—C6—C1	−2.4 (3)	C12—C13—C14—C15	0.4 (3)
C2—C1—C6—O1	−176.98 (18)	C12—C13—C14—N5	−176.46 (18)
C2—C1—C6—C5	1.8 (3)	O3—N5—C14—C15	−178.6 (2)
C6—O1—C7—C8	−172.11 (16)	O2—N5—C14—C15	1.6 (4)
N2—N1—C8—N3	−0.7 (2)	O3—N5—C14—C13	−2.0 (3)
N2—N1—C8—C7	−174.39 (17)	O2—N5—C14—C13	178.2 (2)
C9—N3—C8—N1	1.1 (2)	C13—C14—C15—S2	−0.5 (2)
N4—N3—C8—N1	174.43 (16)	N5—C14—C15—S2	176.31 (17)
C9—N3—C8—C7	174.73 (17)	C12—S2—C15—C14	0.37 (16)
N4—N3—C8—C7	−12.0 (3)	C11—C10—C16—C17	177.31 (17)
O1—C7—C8—N1	−132.7 (2)	S1—C10—C16—C17	−0.9 (3)
O1—C7—C8—N3	54.6 (3)	C10—C16—C17—C22	39.3 (3)
N1—N2—C9—N3	0.8 (2)	C10—C16—C17—C18	−141.3 (2)
N1—N2—C9—S1	−177.89 (13)	C22—C17—C18—C19	−2.3 (3)
C8—N3—C9—N2	−1.18 (19)	C16—C17—C18—C19	178.31 (18)
N4—N3—C9—N2	−174.25 (16)	C17—C18—C19—C20	0.9 (3)
C8—N3—C9—S1	177.59 (13)	C18—C19—C20—C21	0.7 (3)
N4—N3—C9—S1	4.5 (2)	C18—C19—C20—Cl2	179.75 (16)
C10—S1—C9—N2	−155.83 (17)	C19—C20—C21—C22	−0.8 (3)
C10—S1—C9—N3	25.61 (15)	Cl2—C20—C21—C22	−179.87 (15)
C9—S1—C10—C16	135.40 (16)	C20—C21—C22—C17	−0.6 (3)
C9—S1—C10—C11	−42.97 (14)	C18—C17—C22—C21	2.2 (3)
N3—N4—C11—C12	175.41 (15)	C16—C17—C22—C21	−178.45 (18)
N3—N4—C11—C10	−2.5 (3)		

### Hydrogen-bond geometry (Å, °)

**Table:**

*D*—H···*A*	*D*—H	H···*A*	*D*···*A*	*D*—H···*A*
C15—H15A···N2^i^	0.93	2.60	3.495 (3)	162
C21—H21A···Cl1^ii^	0.93	2.81	3.691 (2)	159

Symmetry codes: (i) *x*+1, *y*+1, *z*; (ii) *x*, *y*−1, *z*+1.
